# Prediction of Synergistic Drug Combinations for Prostate Cancer by Transcriptomic and Network Characteristics

**DOI:** 10.3389/fphar.2021.634097

**Published:** 2021-04-12

**Authors:** Shiqi Li, Fuhui Zhang, Xiuchan Xiao, Yanzhi Guo, Zhining Wen, Menglong Li, Xuemei Pu

**Affiliations:** ^1^ College of Chemistry, Sichuan University, Chengdu, China; ^2^ School of Material Science and Environmental Engineering, Chengdu Technological University, Chengdu, China

**Keywords:** prostate cancer, drug combinations, the transcriptomics-based prediction, the network-based prediction, computation

## Abstract

Prostate cancer (PRAD) is a major cause of cancer-related deaths. Current monotherapies show limited efficacy due to often rapidly emerging resistance. Combination therapies could provide an alternative solution to address this problem with enhanced therapeutic effect, reduced cytotoxicity, and delayed the appearance of drug resistance. However, it is prohibitively cost and labor-intensive for the experimental approaches to pick out synergistic combinations from the millions of possibilities. Thus, it is highly desired to explore other efficient strategies to assist experimental researches. Inspired by the challenge, we construct the transcriptomics-based and network-based prediction models to quickly screen the potential drug combination for Prostate cancer, and further assess their performance by *in vitro* assays. The transcriptomics-based method screens nine possible combinations. However, the network-based method gives discrepancies for at least three drug pairs. Further experimental results indicate the dose-dependent effects of the three docetaxel-containing combinations, and confirm the synergistic effects of the other six combinations predicted by the transcriptomics-based model. For the network-based predictions, *in vitro* tests give opposite results to the two combinations (i.e. mitoxantrone-cyproheptadine and cabazitaxel-cyproheptadine). Namely, the transcriptomics-based method outperforms the network-based one for the specific disease like Prostate cancer, which provide guideline for selection of the computational methods in the drug combination screening. More importantly, six combinations (the three mitoxantrone-containing and the three cabazitaxel-containing combinations) are found to be promising candidates to synergistically conquer Prostate cancer.

## Introduction

In the past decades, the drug development has been dominated by one-target one-drug strategy. Although the targeted therapy has dramatically changed the treatment of cancer, confining the drug to a single target fails to consider the complexity of causative factors. Furthermore, cancer cells easily develop resistance to single-drugs through activating other pathways due to their heterogeneous mutation and functional redundancy (Al-Lazikani et al., 2012; Lavecchia and Cerchia, 2016; Liu et al., 2019). Conversely, combinatorial therapies exhibit significant advantages in overcoming drug resistance, reducing toxicity and improving curative effects, thus attracting considerable interests from researchers and drug companies (Bayat Mokhtari et al., 2017; Liu et al., 2019). Considering high attrition rates in the development of new drugs, one tempting option for exploring combinatorial therapies in tumor is to consider drugs already on the market, due to their well-documented safeties (Huang et al., 2019).

In spite of the successes of combinatorial therapies, most of them were derived from the clinical experience and serendipitous discovery, only covering a tiny fraction of the large-scale combinatorial space (Al-Lazikani et al., 2012). In fact, besides more than 200 currently approved cancer agents, there are several thousand drugs under investigation. Consequently, the number of combinations to be tested could reach millions (Ding et al., 2020). It is prohibitively cost and labor-intensive for the experimental approaches to pick out synergistic combinations from the millions of possibilities (Regan-Fendt et al., 2019). Thus, it is highly desired to introduce some effective and robust approaches to significantly narrow down the candidate space of drug combinations for wet-lab experimental validations, in turn facilitating the process of drug synergy prediction.

To mitigate these challenges, various computational methods are coming up recently to assist the combination therapies. Although the predictive ability of these methodologies is significantly better than random, some limitations should be mentioned. Firstly, many existing computational methods (Li et al., 2015; Chen et al., 2016; Regan-Fendt et al., 2019) are based on a similarity comparison between the query combinations and the known ones, thus needing a lot of known drug combinations. However, the number of synergistic combinations known is much less than that of the unknown ones. Secondly, most of the developed predictive models (Zhao et al., 2011; Li et al., 2015; Li et al., 2017; Celebi et al., 2019) require multiple kinds of features, such as physicochemical properties of drugs, interactions between biological entities. In fact, too many features as input would limit the applicability of the method, because the prediction of new drug combination will depend on the same descriptors for each component in the combination (Mason et al., 2018). However, some data may be non-existent or difficult to obtain, in particular for new agents (Chen et al., 2016). In addition, some features may not contribute much to elucidating the underlying mechanisms of drug synergy. As accepted, drug-induced gene expression profiles can be a snapshot of the biological effects induced by drug treatments, thereby benefiting in the recognition of mechanisms of drug action (Lamb et al., 2006; Bansal et al., 2014; Huang et al., 2019). Some studies indicated that gene expression profiles play a significant part in drug predictions (Sun et al., 2015; Celebi et al., 2019; Zhu et al., 2020). Furthermore, there is an growing number of databases which describe biological entities, chemical agents or genomic data and their relationships being produced and available to the public like the Cancer Genome Atlas (TCGA) (Chang et al., 2013) and the Library of Integrated Network-based Cellular Signatures (LINCS) (Subramanian et al., 2017; Keenan et al., 2018; Koleti et al., 2018). The predictive power of transcriptomics-based methods will gain further improvement owing to the availability of such databases. For example, Stathias et al. (Stathias et al., 2018) integrated gene expression data from Cancer Genome Atlas, Library of Integrated Network-based Cellular Signatures, and the Brain Tumor PDX national resource to build a computational platform named SynergySeq in order to identify synergistic combinations in glioblastoma multiforme (GBM). As a result, they identified compounds that synergize with BET inhibitors and further validated their synergistic effects in reducing glioblastoma multiforme cell expansion experimentally. In addition, in the last few years, network-based models were developed to enable researchers to screen synergistic pairs and examine the mechanisms of them, given that both physiological states and biological processes are modulated by a large interactive network with many signaling pathways (Jia et al., 2009; Barabási et al., 2011; Ryall and Tan, 2015; Wu et al., 2018; Cheng et al., 2019; Zhou et al., 2020). For example, according to the approved combinatorial therapies of hypertension and cancer, Cheng et al. (Cheng et al., 2019) quantified the distance between drug targets and disease proteins in the human protein-protein interaction network (PPI), and suggested that a drug combination is effective when meets the criteria of “Complementary Exposure” pattern: the target modules of each drug locates separately within or adjacent to different parts of the disease module. Using hypertension data as a validation set, this network-based predictor achieved 59% accuracy, outperforming traditional cheminformatics and bioinformatics approaches. The work exhibits the role of the network-based information in identifying efficacious combination therapies. However, most of the current models, including the network-based one (Cheng et al., 2019), were constructed using data from various diseases (Bansal et al., 2014; Sun et al., 2015). The models involved in multiple diseases do not take the context specificity into account, while synergy and antagonism have shown to be strongly context-dependent compound-pair properties (Bansal et al., 2014; Yin et al., 2014; Sun et al., 2015). Therefore, it is highly desired to study the context-specific therapies on drug combination prediction.

Prostate cancer (Prostate cancer) has remained an important public health concern since it is the most frequently diagnosed cancer and the second common reason for cancer death in men, which is predicted to have 191,930 new cases and 33,330 deaths in 2020 (Siegel et al., 2020). In 1941, Charles Huggins (Huggins and Hodges, 1941) reported androgen deprivation therapy (ADT) suppressing androgen receptor activity, which has played an important role in treating Prostate cancer. To date, ADT has been used as a standard treatment for Prostate cancer patients. Although ADT exerts certain remissions for 1–2 years for most patients, they still progress to castration-resistant Prostate cancer later, leading to the lethal condition in Prostate cancer. To overcome resistance to monotherapy, some clinical trials like the CHAARTED (Sweeney et al., 2015) and STAMPEDE (James et al., 2015), have shown a survival advantage when combining androgen deprivation therapy with chemotherapy, showing a promise of drug combination in the treatment of Prostate cancer. However, there are only a few approved and investigational drug combinations for Prostate cancer and the success of current Prostate cancer combination therapies are limited (Lee and Kantoff, 2019). Hence, the development of new combinations for Prostate cancer is of great importance.

**FIGURE 1:**
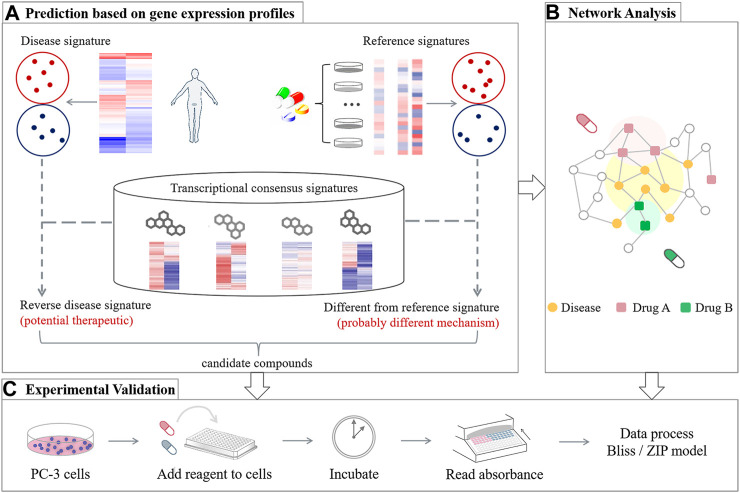
Overview of the design strategy proposed for generating Prostate cancer-specific drug combinations, including three main frameworks: **(A)** Transcriptomics-based ranking, **(B)** network-based assessment, and **(C)** experimental validation.

Inspired by the challenge, we construct a computation-based strategy to screen potential drug combinations for Prostate cancer. Considering high attrition rates in the new drug development, herein, we focus on FDA approved drugs with potential to be repurposed with an existing Prostate cancer single-agent, due to their well-documented safeties. Our prediction framework mainly includes three parts (Figure 1): 1) Transcriptomics-based ranking: computing the synergistic potential for drug pairs by integrating disease transcriptional data with drug-perturbated transcription profiles; 2) Network-based assessment: quantifying the network-based relationship between drug targets of the top ranked pairs and Prostate cancer proteins, in order to assess the predicted drug pairs from a network perspective; and 3) Experimental validation: using cell viability assays to further evaluate the accuracy of the predicted results. The comparison of the two computational results also provides guidelines for selection of the computational methods when applied to a specific disease.

## Materials and Methods

### Collection and Preprocessing of Gene Expression Datasets

In this study, we used two gene expression databases: 1) RNA-Seq data for Prostate cancer tumors and controls were downloaded from Cancer Genome Atlas database (Chang et al., 2013); and 2) the drug-perturbated profiles were downloaded from the Library of Integrated Network-based Cellular Signatures project (Subramanian et al., 2017; Koleti et al., 2018). Specifically, we downloaded the Level 4 panel standardized data from the Phase II L1000 dataset released from the Broad Institute Library of Integrated Network-based Cellular Signatures Data Generation Center through the GEO portal [https://www.ncbi.nlm.nih.gov/geo/query/acc.cgi?acc=GSE70138]. By reannotating all probe sets on different platforms, 12,299 genes shared between the two databases were retained.

### Computation of Disease Signature

Prostate cancer RNA datasets publicly available through Cancer Genome Atlas were downloaded using GenomicDataCommons download tool (Grossman et al., 2016). We obtained 52 prostate cancer and 52 matched normal controls. Then thresholds of |log_2_ *FC*| > 1 and FDR < 0.1 were used to select genes that differentially expressed between tumor and normal samples, leading to the Prostate cancer gene-expression signature.

### Collection and Preprocessing of Gene Expression Datasets

As described in the SynergySeq (Stathias et al., 2018), each compound will be assigned a transcriptional consensus signature (TCS) by utilizing the quantitative gene expression data measured before and after drug perturbation. Then the concordance ratio (CR) and the disease discordance ratio (DR) are calculated for each drug pair (Stathias et al., 2018). Herein, CR denotes the ratio of a compound’s genes in the same direction as the reference signature to those in the opposite directions (Eq. 1). For DR, its definition is based on a comparison between the genes in TSC of a compound and the disease signature genes that are missing in the reference signature. Consequently, DR could be obtained as the ratio of differentially expressed genes induced by drug in an opposite direction to ones in the same direction (Eq. 2):

$$\text{CR}=\frac{\sum_{i=1}^{\text{number}\;\text{of}\;\text{genes}}\left[a_{i}\right]}{\sum_{i=1}^{\text{number}\;\text{of}\;\text{genes}}\left[b_{i}\right]}\;\text{with}\;a_{i}=\{\begin{array}{c} 1,\;if\;z_{i}\cdot r_{i}>0\;\\ 0,\;if\;z_{i}\cdot r_{i}<0 \end{array}\;\text{and}\;b_{i}=\{\begin{array}{c} 1,\;if\;z_{i}\cdot r_{i}<0\;\\ 0,\;if\;z_{i}\cdot r_{i}>0 \end{array},$$ (1)

$$\text{DR}=\frac{\sum_{i=1}^{\text{number}\;\text{of}\;\text{genes}}\left[b_{i}\cdot c_{i}\right]}{\sum_{i=1}^{\text{number}\;\text{of}\;\text{genes}}\left[a_{i}\cdot c_{i}\right]}\;\text{with}\;a_{i}=\{\begin{array}{c} 1,\;if\;z_{i}\cdot d_{i}>0\;\\ 0,\;if\;z_{i}\cdot d_{i}<0 \end{array},\;b_{i}=\{\begin{array}{c} 1,\;if\;z_{i}\cdot d_{i}<0\;\\ 0,\;if\;z_{i}\cdot d_{i}>0 \end{array},\;\text{and}\;c_{i}=\{\begin{array}{c} 1,\;if\;r_{i}=0\;\\ 0,\;if\;r_{i}\neq 0 \end{array},$$ (2)

where z, d, and r denote the TCS vectors of the L1000 compound, the disease and the reference compound signature, respectively.

Combining CR with DR, the orthogonality of each compound to the transcriptional impact caused by the reference compound can be measured by a single value, Orthogonality Score (OS) (Stathias et al., 2018).

$$\text{OS}=\sqrt{{\left(1-\text{CR}\right)}^{2}+{\text{DR}}^{2}}.$$ (3)

### Construction of Human Protein-Protein Interaction Network

Here, we construct a comprehensive human PPI network by using high-quality protein-protein interaction data from different bioinformatics and systems biology databases (Cheng et al., 2019): 1) binary interactions from yeast two-hybrid high-throughputs; 2) binary, physical PPIs derived from protein 3D structures; 3) kinase-substrate pairs; 4) signaling interactions, and 5) literature-curated interactions. As a result, the final human PPI network consists of 217,109 edges and 15,911 nodes.

### Network Configurations of Drug–Drug–Disease Combinations

We assemble Prostate cancer-gene annotation data from eight different bioinformatics data sources: OMIM (Amberger et al., 2015), CTD (Davis et al., 2015), ClinVar (Landrum et al., 2014), GWAS Catalog (Welter et al., 2014), GWASdb (Li et al., 2016), PheWAS Catalog (Denny et al., 2013), HuGE Navigator (Yu et al., 2008), and DisGeNET (Piñero et al., 2015). In addition, we collect the target information of the approved drugs by searching in DrugBank (Law et al., 2014), and drug target interactions meeting three criteria were used (Cheng et al., 2019): 1) binding affinities ≤10 μM; 2) the manually verified target stored in the UniProt with unique identifiers.

In the human PPI network, when a drug targets the corresponding subnetwork of a disease or its adjacent communities, the drug is more likely to have therapeutic effects on the disease than other drugs with targets far from the disease subnetwork (Cheng et al., 2018; Cheng et al., 2019). Z-score is a reliable index to measure the network proximity between a drug (X) and a disease (Y), which is based on the shortest path lengths d(x, y) between drug targets (x) and disease proteins (y):

$$d\left(X,\;Y\right)=\frac{1}{\left\|Y\right\|}\sum_{y\in Y}\min_{x\in X}d\left(x,y\right),\;z=\frac{d-\mu }{\sigma }.$$ (4)

Select a random group of proteins each time, the size and degree distribution of which matches the ones of disease proteins and drug targets, repeat 100 times, and then the mean *µ* and standard deviation *σ* were calculated. If the drug targets and the disease proteins separate from each other from a network-based perspective, their corresponding z ≥ 0; otherwise, z < 0.

In addition, the isolated target protein modules between two drugs in the human PPI network indicating that they act in different ways, and the network-based separation is an effective measurement for this (Menche et al., 2015):

$$s_{AB}=\langle d_{AB}\rangle -\frac{\langle d_{AA}\rangle +\langle d_{BB}\rangle }{2},$$ (5)

where <d> represents the shortest path between two nodes. If the two drug–target modules isolate from each other in the network, their corresponding $s_{AB}\;\geq \;0$ ; otherwise, *s*_*AB*_ < 0.

### Cell Lines and Reagents

PC-3 cells were purchased from the Wuhan Bafeier Biological Co., Ltd. and grown in F12K mediums supplemented with 10% FBS and 1% penicillin-streptomycin at 37 under 5% CO_2_. Docetaxel (D807092) was purchased from Macklin (Shanghai, China). Imatinib (I0906), cabazitaxel (C3390), and mitoxantrone (M3133) were purchased from Tokyo Chemical Industry (TCI, Shanghai, China). Indinavir sulfate (HY-B0689A) and cyproheptadine hydrochloride sesquihydrate (HY-B1165) were purchased from MedChemExpress (MCE, Shanghai, China). MTT (3580MG250) was purchased from BIOFROX (Guangzhou, China).

### MTT Assay

Cells were seeded into 96-well plate at a density of 4.0 × 10^3^ cells/well in growth medium, cultured for 24 h, and then the indicated drugs were added and co-cultured for 72 h. For each concentration gradient, set three replicates, and a well without culture medium was set as control. Then 10 μl MTT solution was added to each well. After 4 h, the cell medium was removed and 150 μl/well of DMSO was added. Relative cell viability was obtained by measuring absorbance at 570 nm in a microplate reader (Flexstation® 3, Molecular Device, United States).

## Results

### Transcriptomics-Based Ranking

In the area of recognizing the mechanisms of human diseases and drug actions, RNA-seq plays a significant role (Lamb et al., 2006; Rees et al., 2016; Wang et al., 2016). Advances in sequencing techniques have generated large-scale omics data, which provide opportunities for drug discovery. As aforementioned, Stathias et al. (Stathias et al., 2018) proposed a method termed SynergySeq to screen drug pairs acting in a synergistic way, in order to combat the resistance of BET inhibitors in glioblastoma multiforme. By assessing the expression of 978 representative landmark transcripts in glioblastoma multiforme and small molecule compounds, they screened some synergistic combinations with a reference compound (BET) from 285 L1000 compounds, and these combinations were further validated by both the external databases and various assays. Herein, we also applied SynergySeq to Prostate cancer. Different from (Stathias et al., 2018), we reannotated the probes on Cancer Genome Atlas and Library of Integrated Network-based Cellular Signatures platforms, resulting in 12,299 common genes, covering a more comprehensive gene space, and the compound library was expanded to 918 approved drugs, much more than 285 in the previous work (Stathias et al., 2018).

#### Compound-Specific Transcriptional Consensus Signatures

In order to predict drug combinations for Prostate cancer, we first need to generate drug-perturbated transcription profiles of Prostate cancer cells from a large-scale database which collects gene expression data before and after drug perturbation across multiple cancer cell lines. For each compound, the Library of Integrated Network-based Cellular Signatures L1000 project provides data on gene expression measured at different time points and doses before and after treatment of multiple cells (Keenan et al., 2018). However, some compounds didn’t treat any Prostate cancer cell lines, so there is a need to establish the respective transcriptional consensus signatures (TCS) for each compound. In the work of Stathias (Stathias et al., 2018), they introduced TCS to represent the transcription profiles of compounds in glioblastoma multiforme cells under the condition that most Library of Integrated Network-based Cellular Signatures L1000 compounds lack profiles from any glioblastoma multiforme cell, and demonstrated that TCSs could represent the compound used to perturb the cells, and be independent of the cell type. Following the work, we calculated TCS for each compound, based on the chemical perturbation experiments data across multiple cell lines. If a gene is observed to be consistently up or down regulated in multiple types of cancer cell lines after the compound disturbance, we deduce that the gene also produces the same transcriptional changes in the prostate cancer cells.

**FIGURE 2:**
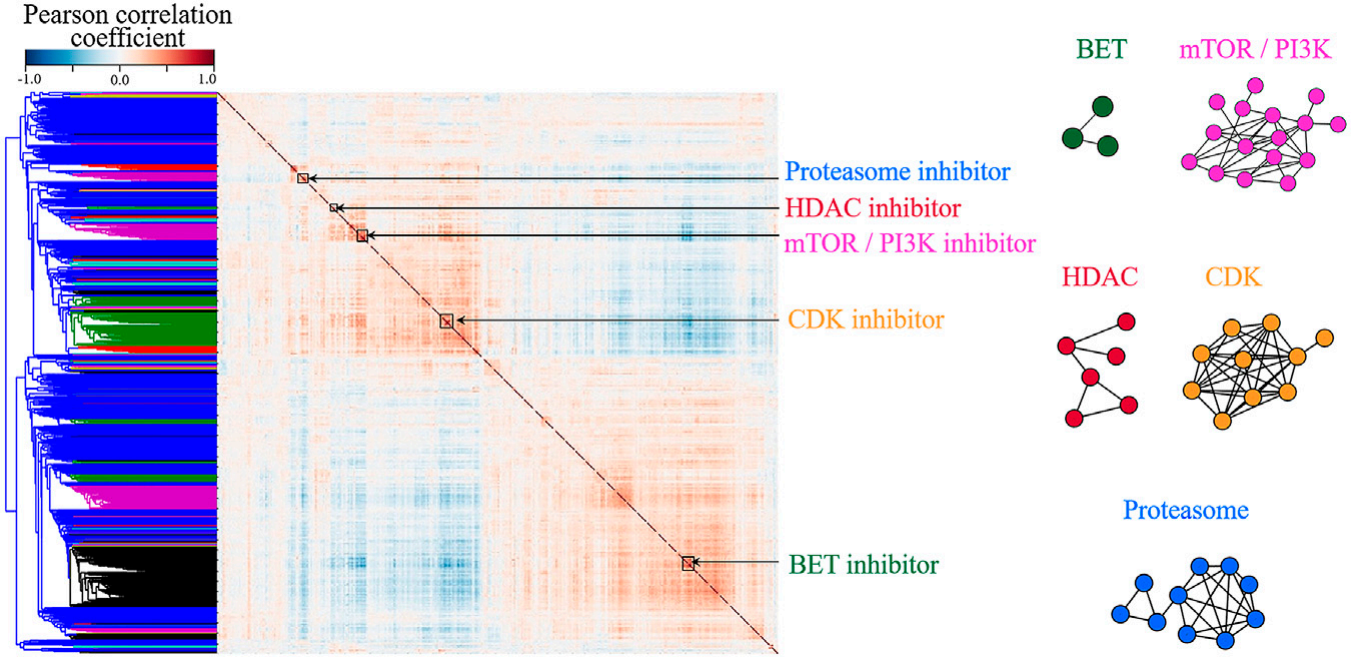
The clustering of small molecules according to their L1000 consensus signatures. **(A)** Correlation matrix of the 918 consensus signatures. Blue to red denotes the correlation coefficient between the two compounds from −1 to 1, namely, from completely negative correlation and the completely positive one. The red clusters along the diagonal denote compounds with high transcriptional similarities (Pearson Correlation > 0.7). **(B)** Networks of highly correlated Library of Integrated Network-based Cellular Signatures compounds. The nodes in the network represent L1000 small molecules. If the correlation coefficient between two compounds is greater than 0.7, they are connected by a line. The color of the network is corresponding to the cluster annotation in **(A)**. For example, blue nodes in **(B)** are Proteasome inhibitors, corresponding to the blue cluster in **(A)**. Compound names and mechanisms of action are shown in Supplementary Table S1.

In order to confirm the assumption, correlation coefficients between all pairs of compounds were calculated using TCS and hierarchical clustering was then performed. As a result, various compound classes are aggregated respectively, as shown in Figure 2 and Supplementary Table S1. Figure 2 shows that compounds with highly correlated consensus signatures (Pearson Correlation > 0.7) could be incorporated into a subnetwork reflecting their mechanism of action, confirming that TCS could well characterize the transcriptomic changes induced by drugs. In addition, the observation further supports the idea that compounds with similar mechanisms produce similar gene expression changes (Lamb et al., 2006; Rees et al., 2016; Regan-Fendt et al., 2019).

#### Reference Compounds

Herein, we need to identify available Prostate cancer drugs from the Library of Integrated Network-based Cellular Signatures L1000 dataset as reference candidates and then repositioned other marketed drugs to find the ones, which could produce synergistic effects with the reference compound selected. For Prostate cancer, only thirteen drugs are selected as preliminary reference candidates in the work, as they are approved drugs for Prostate cancer and also have experimental data for treating Prostate cancer cell lines in the Library of Integrated Network-based Cellular Signatures project. For the thirteen reference compounds, we only focused on genes that induce consistent transcriptional changes in at least half of the PC-3 cells to obtain robust reference signatures (Stathias et al., 2018). Because a high TCS gene score (max score = the number of the cell lines used) indicates that more genes over/under-expressed in different PC-3 cells, three compounds (mitoxantrone, cabazitaxel, and docetaxel), which exhibit significantly higher TCS scores than the other Prostate cancer drugs (vide Supplementary Table S2), are selected as final reference compounds.

In fact, mitoxantrone, cabazitaxel, and docetaxel are all conventional chemotherapeutics to treat Prostate cancer. Mitoxantrone was the only chemotherapeutic drug approved for the treatment of Prostate cancer before 2004. As a DNA intercalating agent and topoisomerase II inhibitor, it has been routinely used for the treatment Prostate cancer since its palliative benefit could enhance clinical remission of the Prostate cancer patients. However, it was also reported that the mitoxantrone failed to confer any survival advantage, and most patients frequently developed therapeutic resistance to the treatment (Song et al., 2018). In 2004, the docetaxel was approved by FDA, which brought certain improvements for the treatment of Prostate cancer patients. Thus, it became the standard chemotherapy treatment for castration-resistant prostate cancer (Song et al., 2018). Unfortunately, many patients did not respond to the therapy and all patients ultimately developed resistance to the docetaxel (Hwang, 2012; Song et al., 2018). Thus, many efforts have been devoted to overcome chemoresistance to docetaxel. Consequently, multiple novel anti-tumor agents were developed, including the cabazitaxel. The cabazitaxel, as the second taxane, could extend survival and is currently used as a single agent (Madan et al., 2011). Despite the antitumor activity of the cabazitaxel in docetaxel-resistance models, cabazitaxel resistance was still proved both *in vitro* and *in vivo* and the resistance mechanisms are still unclear (Natsagdorj et al., 2019; Ylitalo et al., 2020). As known, the cabazitaxel is often administered as a last resort after patients develop resistance to docetaxel. Once the resistance to cabazitaxel is acquired, there are limited therapeutic options. Therefore, it is important to explore the combination therapy based on the three reference compounds to improve survival or clinical outcomes, in turn providing more options for the treatment of Prostate cancer.

#### Prediction of Synergic Effects Based on Transcriptome-Based Data

As accepted, a drug might have the potential to treat a certain disease if its treatment could reverse the gene signature of the disease (Li et al., 2020). Thus, an ideal Prostate cancer drug should has a TCS, which could reverse all abnormally expressed genes in Prostate cancer (Stathias et al., 2018). In the other words, we hope to select the combination of drugs, which could to the largest extent reverse the abnormally expressed genes in Prostate cancer.

Using Cancer Genome Atlas RNA-Seq datasets for Prostate cancer tumors and controls, we identified 1283 differentially expressed genes to comprise the Prostate cancer disease signature. Then we prioritized the compounds based on how much they differ from the reference compounds and how much they reverse the disease signature. First, we calculated CR in terms of Eq. 1, which denotes the overlap between a reference small molecule and the Library of Integrated Network-based Cellular Signatures L1000 one. The higher the CR value is, the more similar the transcriptional responses induced by the two compounds. In other words, they are more likely to target the same disease pathway. Then, DR was estimated by Eq. 2, which gives the reversal degree of disease signature caused by a small molecule different from a reference drug (Stathias et al., 2018). These genes in DR are absent from the Prostate cancer reference signature. The compound has higher DR than the other, suggesting that it has more discordant genes with respect to the Prostate cancer differentially expressed genes. Finally, combining CR and DR, each compound can be scored by a single value (OS; Eq. 3) to quantify its orthogonality to the reference-induced transcriptional effect.

**FIGURE 3:**
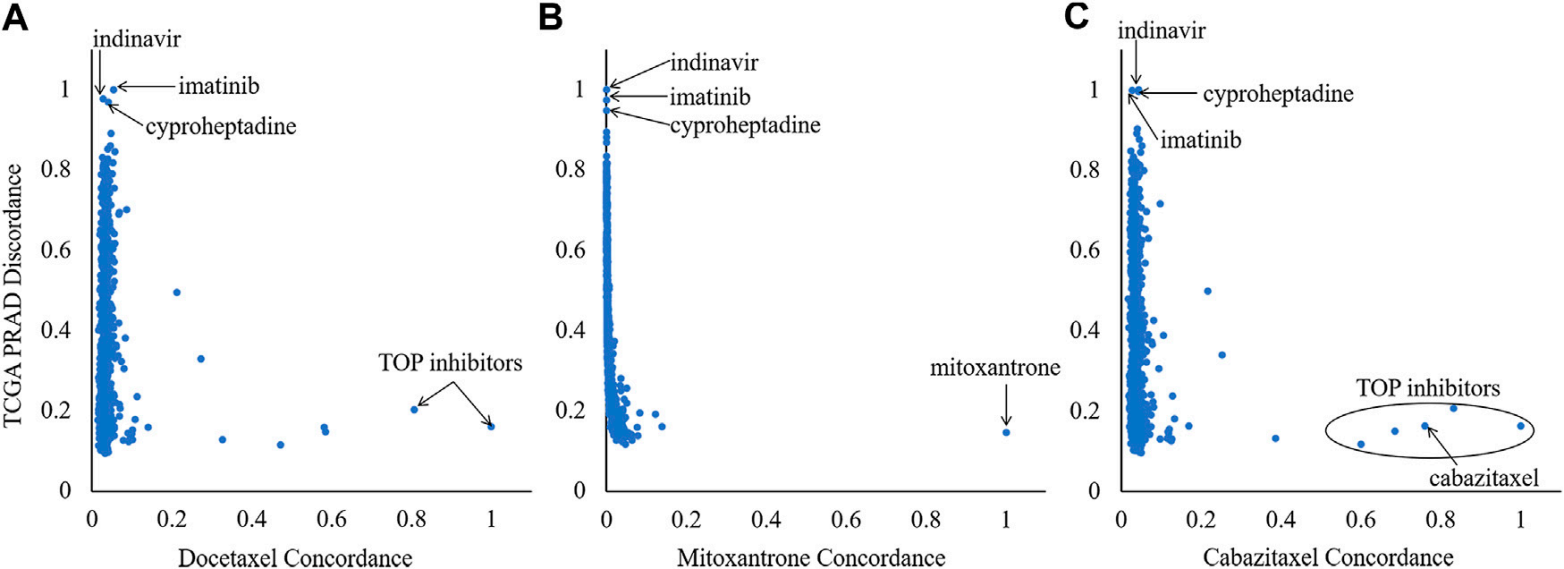
Ranking of the 918 Library of Integrated Network-based Cellular Signatures compounds based on their orthogonality to the signatures of the three references (mitoxantrone, docetaxel and cabazitaxel). *X*-axis and *Y*-axis denote the CR and DR values, respectively.

In order to select compounds with great potential, we did a scatterplot for each reference compound, as shown by Figure 3. According to the criteria that drug pairs with therapeutic effects tend to have high OS, three compounds located in the upper left corner, which have significantly higher OS scores than the others, were selected for each reference compound, leading to nine drug combination candidates. It can be seen from Figure 3 that the top three combined objects are the same for each reference compound, which are indinavir, imatinib and cyproheptadine.

### Network-Based Assessment

It was indicated from the network analysis that a combinatorial therapy is efficacious only when it follows the “Complementary Exposure” pattern, namely, the target modules of each drug in the combination locates separately within or adjacent to different parts of the disease module (Cheng et al., 2019). Therefore, we further constructed the network-based model to assess the drug combinations predicted by the transcriptomics-based method. To achieve this goal, we quantified the network-based relationship between Prostate cancer disease module and two drug-target modules in order to observe if the nine drug combinations fall into the Complementary Exposure category. The results are shown as follows:

For indinavir, the network configuration between it and the three reference compounds are failed to be calculated because the target protein of the indinavir only has Pol polyprotein reported. However, the pol polyprotein is marked as “unreviewed” in the UniProt database. In other words, there is lack of reliable data regarding the target protein for the indinavir so that the network relationship could not be calculated. This is a limitation for application of the network analysis in practice, which requires specific target proteins.

**FIGURE 4:**
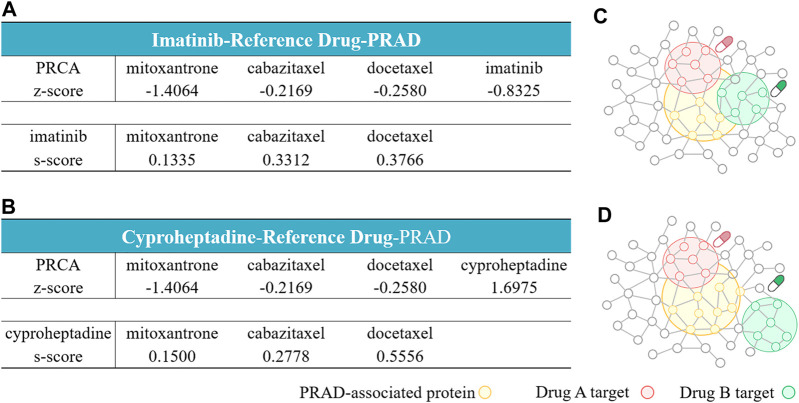
Network configurations of drug–drug–disease combinations. **(A, B)** The network-based relationship between two drug–target modules and one disease module on imatinib-reference drug- Prostate cancer **(A)** and cyproheptadine-reference drug-Prostate cancer **(B)**. **(C, D)** The exposure mode of the Prostate cancer-associated protein module to the pairwise drug combinations: the three imatinib-containing combinations **(C)**, and the three cyproheptadine-containing combinations **(D)**. The z-scores measure the drug–disease separation. The s-scores denote the topological relationship between two drug target modules.

For the imatinib-containing combinations, our network analysis shows that imatinib and the three reference compounds all target different parts of the Prostate cancer-related subnetwork by “Complementary Exposure” pattern. Specifically, the relative proximity between the four drugs (imatinib and three reference compounds) and the Prostate cancer module is negative, z < 0, suggesting that the drug target modules in the combination overlap with the disease module. In addition, the network proximity between imatinib and the three reference compounds is positive (*s*_*AB*_ ≥ 0), indicating that the two drug targets are topologically separated. Thus, the network analysis further supports that the imatinib-containing combinations may be potential for the treatment of Prostate cancer (Figure 4A), in line with the prediction of the transcriptomics-based analysis above.

For the cyproheptadine-containing combinations, although the cyproheptadine hits different targets from the reference compound (*s*_*AB*_ ≥ 0), it failed to hit the disease module (z > 0). Cheng reported that the efficacy of the combinatorial therapy isn’t better than the single-agent therapy if at least one agent locates far from the disease subnetwork (Cheng et al., 2019). Judged from the network-based result, the cyproheptadine-containing combinations should be ineffective for Prostate cancer, which is opposite to the transcriptomics-based prediction.

### Experimental Validation of the Predictions

As observed above, there are some discrepancies between the transcriptomics-based prediction and the network-based one. Thus, we further used the experimental method to validate the prediction results from the two methods. Herein, we used the MTT assay, which is a popular tool in measuring the metabolic activity of living cells, to estimate the cytostatic effects of a monotherapy or a combination of them on PC-3 cells (vide Supplementary Table S3). In order to assess the degree of synergy or antagonism, the combined effects of a drug pair are usually compared to the theoretically expected values using a reference mode, with the assumption that there is no interaction between the components of the combination. The reference models employed here are Bliss (Bliss, 1939) and ZIP (Yadav et al., 2015) models, which are implemented in SynergyFinder web-application (https://synergyfinder.fimm.fi; ref (Ianevski et al., 2017)).

**FIGURE 5:**
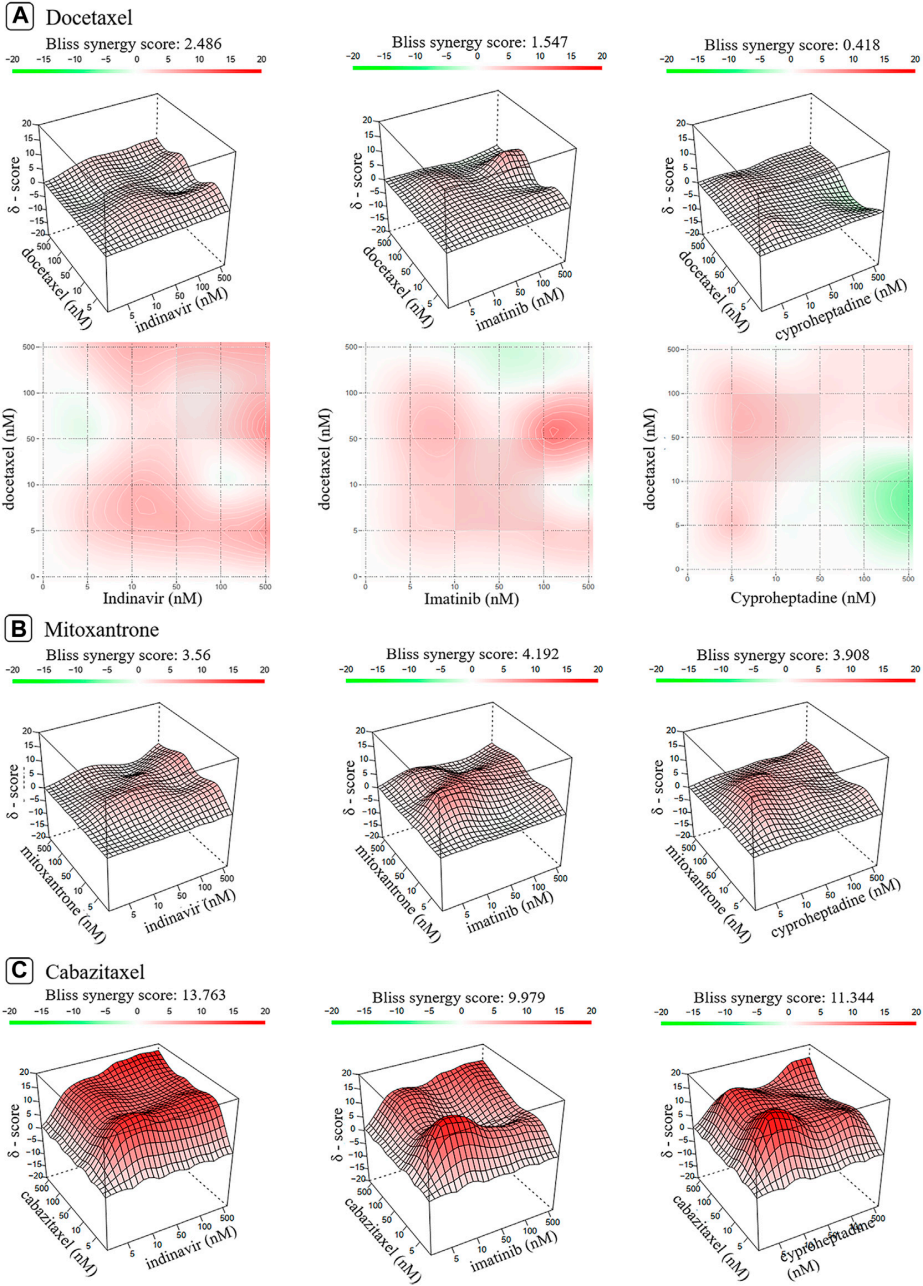
The 2D and 3D heat maps of the combination responses for docetaxel-containing **(A)**, mitoxantrone-containing **(B)** and cabazitaxel-containing combinations **(C)** according to Bliss model. Red represents the synergistic interaction while green denotes the antagonistic interaction.

Synergy scores are listed in Table 1, which are derived from the dose-matrix combinations. Figure 5, Supplementary Figures S1, S2 show the 2D and 3D synergy heat maps for the Bliss and ZIP models of the interactions, through which the combined effects of the nine drug combinations against PC-3 cells (vide) could be obtained. The results shown in Figure 5A revealed that the docetaxel-containing combinations inhibit PC-3 cell proliferation in a dose-dependent manner. In other words, the combination could produce different effects on PC-3 cells due to the different concentration of its components, including antagonistic, addictive, or synergistic effects. Specifically, there is antagonism between the docetaxel and the indinavir when the concentration of the docetaxel is between 40 and 500 nM. The combination of the docetaxel and the imatinib also exhibits antagonism when the concentration of the docetaxel is higher than 100 nM. For the docetaxel-cyproheptadine, this pair presents antagonism when concentration of indinavir is lower than 50 nM, and cyproheptadine is greater than 50 nM. While at other range of drug doses pairs, the docetaxel-containing combination induces additive/synergistic effects. Different from the dose-dependent effects of the combinations containing docetaxel, the three combinations containing mitoxantrone all show overall synergistic effects within the experimental dose range, judged from Figure 5B). In addition, it can be observed from Figure 5C that the three combinations containing cabazitaxel show the strongest synergistic effects. In a whole, the experimental results almost support the transcriptomics-based predictions, but exhibit some discrepancies with the network-based predictions for the mitoxantrone-cyproheptadine and cabazitaxel-cyproheptadine.

**TABLE 1:** Synergy scores for each drug combination according to Bliss model.

Drug combination	Synergy score	Most synergistic area score	Model
docetaxel-indinavir	2.49	3.11	Bliss
docetaxel-imatinib	1.55	2.66	Bliss
docetaxel-cyproheptadine	0.42	1.61	Bliss
mitoxantrone-indinavir	3.56	5.28	Bliss
mitoxantrone-imatinib	4.19	5.88	Bliss
mitoxantrone-cyproheptadine	3.91	5.63	Bliss
cabazitaxel-cyproheptadine	13.76	15.03	Bliss
cabazitaxel-imatinib	9.98	10.63	Bliss
cabazitaxel-cyproheptadine	11.34	13.66	Bliss

## Discussions

Drug combinations play a major part in combating various complex diseases due to increased therapeutic efficacy, decreased toxicity and counter drug resistance. And computational methods bypass the combinatorial explosion problem by greatly reducing the search space and prioritizing combinations. Among these approaches, the transcriptomics-based and the network-based methods have attracted much attention and achieved remarkable performance. In addition, most existing methods focused on multiple diseases. However, the drug synergy is a strongly context-dependent property. Thus, it is highly desired to explore disease-specific synergy combinations. Prostate cancer is a primary factor of male morbidity and mortality, and the inevitable drug resistance to exiting monotherapies highlights the need of new combination therapies. Therefore, we hope to establish a synergistic drug prediction model for Prostate cancer in the work. We firstly employ a transcriptomics-based approach to reposition 918 approved drugs in combination for Prostate cancer, through which the nine synergistic combinations are identified. To compare the performance of different computational methods to predict the drug pair of Prostate cancer, we further utilize the network-based method proposed by Cheng et al. (Cheng et al., 2019) to assess the synergistic potential of the six drug combinations from the transcriptomics-based prediction, excepting for the three indinavir-containing combinations, since the indinavir lacks reliable drug targets. The network-based results show that the three imatinib-containing combinations fall into the Complementary Exposure category with the Prostate cancer disease module. Thus, the network-based method show that they are effective combinations for Prostate cancer, in line with the transcriptomics-based prediction. However, the three cyproheptadine-containing combinations are predicted as nonsynergistic combinations, which conflicts with the transcriptomics-based results. To validate the computational results, we further conduct *in vitro* experiments. The *in-vitro* results show that the combined effects of the three docetaxel-containing combinations are in a dose-dependent manner while the other six combinations could synergistically inhibit the growth of PC-3 cells, thus supporting the transcriptomics-based predictions. However, the two combinations (cyproheptadine-mitoxantrone and cyproheptadine-cabazitaxe), which are predicted to be nonsynergistic by the network-based method, present strongly synergistic in the *in-vitro* experiment instead. Only the network-based predictions of imatinib-mitoxantrone and imatinib-cabazitaxel are consistent with the *in-vitro* results.

Specifically, the two imatinib-containing combinations (mitoxantrone-imatinib and cabazitaxel-imatinib), which are consistently predicted as synergistic combinations in the transcriptomics-based and the network-based analysis, are further proved *in vitro* and exhibit highly potential for the combination therapy of Prostate cancer. In fact, it is revealed that imatinib could inhibit PDGFR, a potential therapeutic target in Prostate cancer (Pinto et al., 2012). Unfortunately, the efficacy of single-agent PDGFR inhibitors in patients with metastatic Prostate cancer appears limited. Interestingly, it was observed that combining imatinib with other anticancer drugs might increase the effectiveness of the single-agent PDGEF inhibitor (Kim et al., 2012). Moreover, the imatinib was found to decrease interstitial fluid pressure in solid tumors so that it could improve tumor delivery of anticancer drugs *in vivo* (Vlahovic et al., 2007). All the evidences also provide further support for the potential of the imatinib in combination therapy for Prostate cancer. The two cyproheptadine-containing combinations (mitoxantrone-cyproheptadine and cabazitaxel-cyproheptadine), which are predicted to be synergistic combinations in the transcriptomics-based prediction but nonsynergistic in the network-based analysis, show to inhibit the proliferation of PC-3 cells synergistically *in vitro*. Although the therapeutic effect of cyproheptadine in Prostate cancer has never been reported, the use of cyproheptadine in the treatment of multiple malignancies, such as myeloma, leukemia and hepatocellular carcinoma (Rosenberg and Mathew, 2013), to some extent suggest the effect of cyproheptadine in combating cancers. Therefore, it is reasonable for the two cyproheptadine-containing combinations to be potential for the Prostate cancer treatment. The two indinavir-containing combinations (mitoxantrone-indinavir and cabazitaxel-indinavir), which failed to conduct the network-based analysis, also were experimentally confirmed to be synergistic combinations. Indinavir is a human immunodeficiency virus protease inhibitor (HIV PIs), which was proved *in vitro* and *in vivo* to slow down the proliferation, promote the apoptosis and inhibit the growth of tumor cells (Toschi et al., 2011; Barillari et al., 2014; Maksimovic-Ivanic et al., 2017). The anti-tumor activity of HIV PIs has also reported in many studies on treating tumors like Kaposi’s sarcoma, lymph-gland tumor, or Prostate cancer (Toschi et al., 2011; Barillari et al., 2014; Maksimovic-Ivanic et al., 2017). In addition, the CYP3A4 participates in the process of metabolism and the development of resistance (Ikezoe et al., 2004; Van Eijk et al., 2019), while indinavir as a potent inhibitor of CYP3A4 is thought to enhance the therapeutic effects of anticancer drugs in androgen-independent prostate cancer cells.

Judged from the experimental results, the transcriptomics-based predictor performs better than the network-based analysis, at least for Prostate cancer One reason may be that this network approach used in the work is based on the analysis of hypertension and pan-cancer data while the models built on data from a variety of diseases are more likely to miss some important features that being beneficial for capturing unique combinations with therapeutic effectiveness for a specific disease like Prostate cancer (Sun et al., 2015). As an attempt, we reconstructed a tissue specific Prostate interactome. Specifically, we first calculated the median expression of each gene in the tumor or normal samples from the prostate tissue, after downloading gene expression and phenotype data of Cancer Genome Atlas (Chang et al., 2013) and GTEx (Consortium, 2013). And then, proteins with median expression >1 Transcripts Per Million (TPM) (Sriram et al., 2019) were screened, which are considered to express in the prostate commonly. Finally, the full human PPI network is narrowed down to a subnetwork specific to the prostate, with 214,351 edges and 15,784 nodes. Then, we calculated the configurations of the six drug-drug- Prostate cancer combinations (vide Supplementary Figure S3) are the same as those obtained by the full network calculation (vide Figure 4). The result implies that it may be difficult for the network topology to capture the characteristic of the specific disease. In contrast, the information from the transcriptional level of the specific disease (Prostate cancer) could reflect individual characteristics. In addition, as proposed by Cheng (Cheng et al., 2019), some factors, the incompleteness of the human PPI network and the limited knowledge of proteins associated with the disease and drugs, may impose restrictions on the performance of the current network-based approaches used to develop therapeutic strategies. For example, some drugs have no target proteins available for the network calculation like the indinavir under study. Therefore, researchers should be more cautious when purely using network-based methods to predict drug combinations for a specific disease.

Also, it is noted that the reference signatures used in the transcriptomics-based model is derived from perturbational gene-expression data on PC-3 cell lines, which may not match the disease signature perfectly. But, only the PC-3 cell-line is disturbed by all the 13 approved prostate cancer drugs in the GSE70138 dataset while the LNCAP one is disturbed by one drug (mitoxantrone). For the DU-145 cell-line, there is no profiles induced by any of the 13 drugs. As known, the PC-3 cell-line is derived from metastatic prostate cancer and has been served as standard cell in the drug research on the prostate cancer. To maintain the consistency between the calculation and the experiment, we validated the predictive combinations by *in vitro* experiment only on the PC-3 cells. Additionally, we did a computational comparison. We used the gene expression profiles of LNCAP cells induced by the mitoxantrone to perform transcriptome-based predictions. As shown by Supplementary Figure S4, the three candidates (imatinib, indinavir and cyproheptadine) also rank the top, in line with the prediction from the PC-3 cell, implying to some extent consistency between the two cell-lines. In fact, many drug prediction models are also based on pan-cancer data without considering cancer types due to the limited data available for each cancer type, but they still achieved satisfactory performance when applied to specific cancer (Geeleher et al., 2014; Sun et al., 2015; Cheng et al., 2019), implying that there are some features shared across different cancers, which contribut to drug predictions. In this study, we only studied one cancer type (i.e., Prostate cancer). Although the patient samples and cancer cells belong to different stage, it should be reasonable to assume that there are some characteristics shared between different stages of the prostate cancer. In addition, it is found from some previous literatures (Mao et al., 2008; Kim et al., 2012; Barillari et al., 2014; Hsieh et al., 2016; Sun et al., 2016; Takemoto et al., 2016; Maksimovic-Ivanic et al., 2017; Van Eijk et al., 2019) that the three drugs (i.e., indinavir, imatinib, and cyproheptadine) predicted as one component of the drug combination exhibited anticancer impact on various cancers, including prostate cancer, which also to some extent supports the rationality of using perturbational gene-expression data on PC-3 cell lines to predict drug combinations for the prostate cancer. However, more *in vitro* and *in vivo* experiments will be needed to further validate the therapeutic efficacy of our predictive drug combinations for the prostate cancer.

Despite fascinating advantages of combination therapies, there are still some limitations and challenges needed to be addressed. Firstly, using multiple drugs in the combination may precipitate undesired side effects, and make it difficult to identify which drug is responsible for the effects (Kavanagh et al., 2018). Secondly, the determination of drug dose and ratio in combination therapy is much more complicated than that of monotherapy, because the solubility, stability, pharmacodynamics, and pharmacokinetics of different drugs may vary greatly (Sun et al., 2016). In addition, most of the existing drug combination predictive models are based on omics and drug response data. There has been lack of sufficient data to model the unique characteristics of patients (Kening et al., 2020), which is a major limitation of current researches, including our study. Overcoming these limitations will further increase the value of combination therapies, which requires the joint efforts of researchers across various disciplines, such as biology, chemistry, medicine, and computer science.

## Conclusions

In summary, our results show that the transcriptomics-based strategy is more suitable for the specific disease than the network-based one, at least for Prostate cancer, which will assist in decision making for the usage of the computation methods in the drug combination prediction. More importantly, six drug combinations (i.e., the three mitoxantrone-containing and the three cabazitaxel-containing combinations) are found to be potential to synergistically conquer prostate cancer, which offer promising candidates for preclinical testing. Despite the encouraging results, our findings still require further preclinical testing and clinical trials.

## Data Availability

All datasets generated for this study are included in the article/Supplementary Material. The source code used in this study is available at https://github.com/shiqili-17/drugpair_prediction.
